# Removal of the Homolog Tellurium of Polonium by SiO_2_ Nanofiber Filter for Lead Alloy-Cooled Reactors

**DOI:** 10.3390/toxics10060275

**Published:** 2022-05-24

**Authors:** Xujie Chen, Xiyong Chen, Xian Zeng, Yuan Zhao, Xiaoping Li, Xi Huang, Toyohisa Fujita, Xinpeng Wang

**Affiliations:** 1 School of Resources, Environment and Materials, Guangxi University, Nanning 530004, China; chanxj00@163.com (X.C.); xiyongchen@gxu.edu.cn (X.C.); Pu976811@163.com (X.L.); fujitatoyohisa@gxu.edu.cn (T.F.); 2 China Nuclear Power Technology Research Institute, Shenzhen 518000, China; zengxian@cgnpc.com.cn (X.Z.); zhaoyuan2@cgnpc.com.cn (Y.Z.); 3 Advanced Nuclear Energy Research Group, College of Physics and Optoelectronic Engineering, Shenzhen University, Shenzhen 518060, China

**Keywords:** lead–bismuth eutectic, polonium, SiO_2_ nanofiber filter, density function theory

## Abstract

The lead–bismuth eutectic (LBE) can be easily activated by neutron radiation to produce the radionuclide ^210^Po. It is therefore necessary to establish an effective method to remove vaporized polonium in the cover gas to prevent its release into the air in scenarios of reactor maintenance and coolant leakage accidents. This paper presents a SiO_2_ nanofiber membrane prepared based on the electrostatic spinning and calcination process. The SiO_2_ nanofiber membrane had the advantages of good flexibility, high-temperature resistance, and corrosion resistance. In the trapping experiments, the SiO_2_ nanofiber membrane filters showed excellent filtration performance at 300~400 °C, and the filtration efficiencies for Te, Pb, and Bi could reach 99%, 99%, and 98%, respectively. Proper filtration temperature and gas flow rate are important to maintain high filtration efficiency. After five cycles, the SiO_2_ nanofiber membrane filter still exhibited excellent cycle-use performance. In the density functional theory (DFT) calculations, PbPo and PbTe had strong interactions with amorphous SiO_2_, having adhesion energies of −2.96 to −2.83 eV/molecule.

## 1. Introduction

With the advantages of a low melting point, high boiling point, small neutron cross-section, and high thermal conductivity, lead–bismuth eutectic (LBE) has become a spallation target material and a coolant for accelerator-driven subcritical systems (ADSs) [1,2,3,4]. However, LBE can be easily activated by neutron radiation to produce the radionuclide ^210^Po, which is a highly toxic radionuclide with a physical half-life of 138.4 days. If the cumulative radiation dosage reaches 4 MBq/kg-body-mass in a short time, it will be fatal to the human body. Therefore, to achieve the wide use of LBE in nuclear energy applications, an effective Po pollution prevention system must be established [5,6]. At present, polonium in molten LBE can be effectively removed by lead–polonium distillation, Po-hydride separation, rare-earth filtration, and alkaline extraction [7,8,9]. However, these methods are not applicable for removing polonium evaporated from liquid LBE. Without any prevention means, polonium on the surface of the liquid LBE and its vapor in the cover gas will diffuse out of the main system and be released into the atmosphere during reactor maintenance and coolant leakage accidents [10,11,12,13]. Therefore, removal of polonium from the gas phase needs to be seriously examined.

In an ADS system, the cover gas typically will have the highest concentration of polonium [14,15]. To reduce the concentration of polonium in the cover gas, efforts have been made to carry out the purification of gaseous polonium compounds and aerosols in the lead stack cover gas. Obara et al. used a stainless-steel mesh filter to remove polonium aerosols in the gas phase under vacuum conditions, and the filtration effect was significant [16,17]. However, the filtration performance of the stainless-steel mesh filter causes a severe decrease or even ineffectiveness due to the corrosion behavior of stainless steel in LBE [18]. Based on thermal chromatography experiments, a high adhesion affinity of polonium was achieved using noble metals (Au, Ag, Pt, and Pd) and 316L stainless steel, as reported by Maugeri et al. [19,20,21]. Meanwhile, the adhesion energies of Po, Po_2_, PbPo, PbBi, H_2_Po, and PoOH on noble metals were calculated by using the density functional theory (DFT) for ^210^Po trapping [22,23,24]. However, the high price of noble metals limits its large-scale application for polonium removal. The development of polonium aerosol filtration materials with high-temperature resistance, corrosion resistance, and high filtration accuracy is still the main focus of research [25].

Nanofibers with small pore size, high throughput, and high filtration accuracy are regarded as candidate filtration materials in the field of nuclear waste treatment [26,27,28]. According to the literature, the whole process of evaporating and capturing volatile materials involves sub-processes of volatilization, condensation, and solidification [29,30,31], which are also applicable for the current study. Polonium-containing vapors migrate from liquid lead–bismuth pools to the cover gas at high temperatures. The supersaturated vapors will thereafter undergo two processes to form aerosol particles: spontaneous nucleation (vapor condensation in the gas phase to form new particles) and nonspontaneous nucleation (vapor condensation and deposition directly on impurity particles). As for aerosol particles, the nanofibers capture these particles by inertial collision, interception, Brownian motion, and gravitational sedimentation [32]. Inorganic nanofibers meet the requirements of high-temperature resistance, corrosion resistance, and high filtration accuracy of polonium filters, and they remove gaseous components and aerosols in the gas phase through condensation and physical filtration to achieve the purification of the cover gas in a lead-based reactor [33]. However, most of the inorganic nanofibers have limited applications in gas purification due to their own brittleness.

Electrospun fibers with small fiber diameter, large aspect ratio, and high porosity have outstanding filtration and barrier properties. Moreover, the SiO_2_ material itself has the characteristics of high temperature and corrosion resistance. In this study, the SiO_2_ inorganic nanofiber membranes prepared based on electrostatic spinning have excellent flexibility and overcome the disadvantage of brittle inorganic nanofibers, which can be used for the removal of polonium from the cover gas in LBE under high-temperature conditions [34,35]. For the sake of safety in the lab experiments, Te was used in this study as a substitute for the Po radionuclide, which has been proven to be feasible in previous published works [36,37]. In particular, this work focused on the filtration performance and mechanism of the inorganic nanofiber membrane for Te aerosol in the gas. The results provide practical insights for removing Po aerosols in the cover gas in a real ADS system. Furthermore, calculations based on DFT were applied to study the adhesion energies of possible adsorbate molecules (Po, Te, Bi, PbPo, and PbTe) on the surface of amorphous SiO_2_ to explain the possible interaction mechanisms. The proposed SiO_2_ nanofiber filter has outstanding filtration performance for the removal of polonium under high temperature and airflow conditions and cycle-use performance, so it has the potential to be used in the polonium purification systems.

## 2. Materials and Methods

### 2.1. Materials

All chemicals used in this study were of analytical grade and were directly used without further treatment. Phosphoric acid (H_3_PO_4_, 85 wt.%) was purchased from Xilong Chemical Co., Shantou, China and nitric acid (HNO_3_), tetraethyl orthosilicate (TEOS), and poly(vinyl alcohol) (PVA1788) were purchased from Sinopharm Chemical Reagent Co., Ltd., Shanghai, China.

### 2.2. Preparation of SiO_2_ Nanofiber Membrane

The SiO_2_ inorganic nanofibers used in the filter were prepared by electrostatic spinning and high-temperature calcination processes. Tetraethyl orthosilicate (10.4165 g), deionized water (9 g), and phosphoric acid (0.577 g) were first mixed and stirred at room temperature for 12 h to obtain a silica sol after full hydrolysis. PVA powder was dissolved in deionized water and stirred in a water bath at a temperature of 90 °C for 4 h to prepare a 10 wt.% PVA solution. The prepared silica sol and the PVA solution were later mixed at a mass ratio of 7:3 and stirred for another 12 h to obtain an electrostatically spun precursor solution. The prepared precursor solution was thereafter loaded into a syringe and fixed on an electrostatic spinning device (Electrospinning Equipment, ET-2535DC).

During the electrospinning process, the charged polymer droplets are accelerated at the apex of the Taylor cone at the syringe needle under the action of an electric field force. When the electric field force is large enough, the polymer droplets overcome the surface tension to form a jet stream. The solvent evaporates or solidifies during the trickle jetting process and finally falls on the receiving device to form a non-woven nanofiber mat [26]. In the current experiments, the electrospinning process parameters were set as follows: the temperature was 40–50 °C, the humidity was 30–40%, the receiving distance was 17 cm, the injection speed was 1 mL/h, and the accelerating voltage was 8 kV. The obtained electrospinning precursor nanofiber membrane was dried in an oven at 80 °C for 6 h. The dried nanofiber membrane was later calcined in a muffle furnace at 800 °C for 2 h to remove the organic matter, thereby yielding flexible SiO_2_ inorganic nanofibers. The corresponding specific surface area and the average pore size were generally about 5.39 m^2^/g and 15.50 nm, respectively, for all the prepared nanofiber membrane samples, which were determined using the method described in the next section.

### 2.3. Phase and Microstructure Characterization

The crystal structures of the prepared pure SiO_2_ nanofibers before and after the Te trapping experiments (described in Section 2.4) were analyzed by X-ray diffraction (XRD, Rigaku D/MAX 2500V). The N_2_ adsorption–desorption curves of the SiO_2_ nanofibers were measured by a Tristar II 3020 instrument. The specific surface areas were calculated according to the Brunauer–Emmett–Teller (BET) method, and the pore size distribution was derived from the desorption branch of the isotherm with the Barrett–Joyner–Halenda (BJH) model. Prior to the test, the samples were treated under vacuum for 12 h at 200 °C. The surface morphologies and compositions of the intact SiO_2_ nanofibers and the LBE-Te@SiO_2_ samples obtained after the Te trapping experiments were analyzed by scanning electron microscopy (SEM, Hitachi SU5000, Tokyo, Japan) and energy-dispersive X-ray spectroscopy (EDS).

### 2.4. Te Trapping Evaluation Setup and Procedure

**Figure 1:**
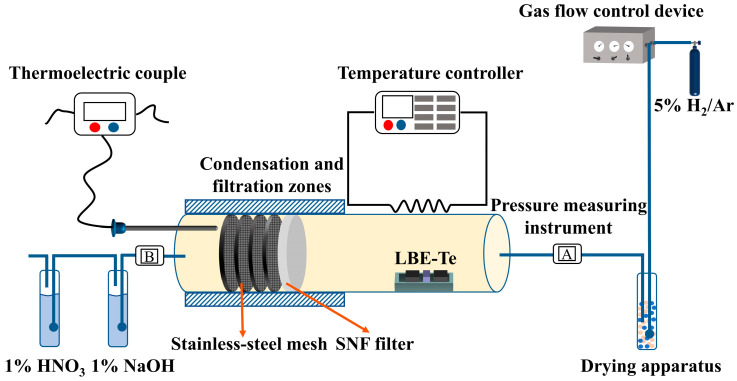
Schematic diagram of aerosol capture system.

The Po evaporation in the lead–bismuth fast reactor was simulated by replacing the radioactive Po with the light element Te within the same main group. Figure 1 shows a schematic of the experimental system that was designed to capture Te with an SiO_2_ nanofiber filter (SNF) under gas flow. The SNF, which was composed of a flexible SiO_2_ inorganic nanofiber membrane and a stainless-steel mesh and fixed in a stainless-steel sealing ring, was seamlessly installed in the stainless-steel tube. Sufficient layers of the stainless-steel mesh were adopted in the current study to allow complete capture of the compounds of interest. A quartz boat lined with asbestos was preloaded with the volatile sample of LBE-Te (2.85 g of LBE with 0.15 g of pure Te) and placed in the volatilization zone of the stainless-steel tube. The air-exposed part of the stainless-steel tube on the filtering side was insulated with asbestos, and a thermocouple was used to measure the filter temperature in real time. The pressures at the gas inlet and outlet were recorded with pressure-sensing gauges. During the experiment, a reducing atmosphere of 5 wt.% H_2_ balanced with Ar was applied as the carrier gas. The flow rate of the carrier gas was controlled with a gas flow controller. Before heating up the LBE-Te sample, the test system was pre-fluxed with the carrier gas for at least 15 min. After all the air in the test tube was replaced with the carrier gas, the volatilization zone was rapidly heated to 690 °C at a rate of 15 °C/min. The trapping experiment was sustained for 5 h after the heating temperature reached 690 °C. The filtered waste gas was discharged after passing through the acid–base tail gas removal device. Table 1 lists the experimental conditions used in this study. The set I experiments explored the influence of the filter temperature on the filtration efficiency of the SNFs for Te, Pb, and Bi. The set II experiments examined the effects of the loading level of the SiO_2_ filter. The set III and IV experiments were designed to test the flow rate of the carrier gas for the influence of filtration efficiency. The cycle-use performance of the SiO_2_ filter was studied by the set V experiment.

### 2.5. Evaluation of Filtration Efficiencies of SiO_2_ Nanofiber Membrane Filters

The Te evaporated from the LBE in the current experimental setup could be captured by the SiO_2_ nanofiber membrane, collected by the stainless-steel mesh installed after the SiO_2_ membrane, and deposited on the stainless-steel tube wall before the filtration membranes. The Te filtration efficiency *η* by the SNFs can be evaluated with the following equation (Equation (1)):

(1) $$\eta =m_{1}\;/\;\left(m_{1}+m_{2}\right)\times 100\%,$$

where *m*_1_ and *m*_2_ are the corresponding Te masses captured by the SiO_2_ nanofiber membrane and the stainless-steel mesh, respectively. This equation is also applicable for evaluating the filtration efficiency for the evaporated Pb and Bi in the cover gas.

In engineering practice, the filtration factor (QF) has become a meaningful index to identify the filtration performances of materials to balance the filtration efficiency and flow resistance. QF is defined as follows (see Equation (2)):

(2) $$\mathrm{QF}=\frac{-\ln\left(1-\eta \right)}{\Delta p},$$

where Δ*p* is the pressure drop through the membrane, and *η* is the filtration efficiency.

To obtain the masses of the captured element of interest, the SiO_2_ nanofiber membrane and stainless-steel mesh after the experiments were immersed in a 7 mol/L nitric acid solution and ultrasonically oscillated to obtain a solution of Te, Pb, and Bi ions. The ion concentrations in the solution were determined by using an inductively coupled plasma emission spectrometer (ICP) to evaluate the corresponding mass deposition of the volatile elements in both the SiO_2_ nanofiber membrane and the stainless-steel mesh.

### 2.6. Density Functional Theory Calculations

The calculations were performed on the DFT framework of the Gaussian 16 program package. The SiO_2_ surfaces were represented using Si_4_O_4_(OH)_9_ clusters. The terminal oxygen atoms were saturated with hydrogen atoms [38,39]. First, geometry optimization calculations were performed with the three-parameter hybrid functional (B3LYP) [40]. The geometry optimizations of the Si, O, and H atoms were performed with the standard 6-311G(d) basis set, and the geometry optimizations of the Pb, Po, Te, and Bi atoms were performed with the SDD basis set. Finally, the energy calculations of the optimized structure were based on the def2-TZVP basis set. The adhesion energy was calculated to analyze the direct interactions between molecules and the surface oxygen defects based on the following equation (Equation (3)):

(3) $$E_{\mathrm{ads}}{=E}_{\mathrm{AB}}-{\;E}_{A}-{\;E}_{B},$$

where *E*_ads_ is the adhesion energy, and *E*_AB_, *E*_A_, and *E*_B_ are the energies of the complex, monomer A, and monomer B, respectively.

## 3. Results and Discussion

### 3.1. Phase and Structural Analysis of LBE-Te

**Figure 2:**
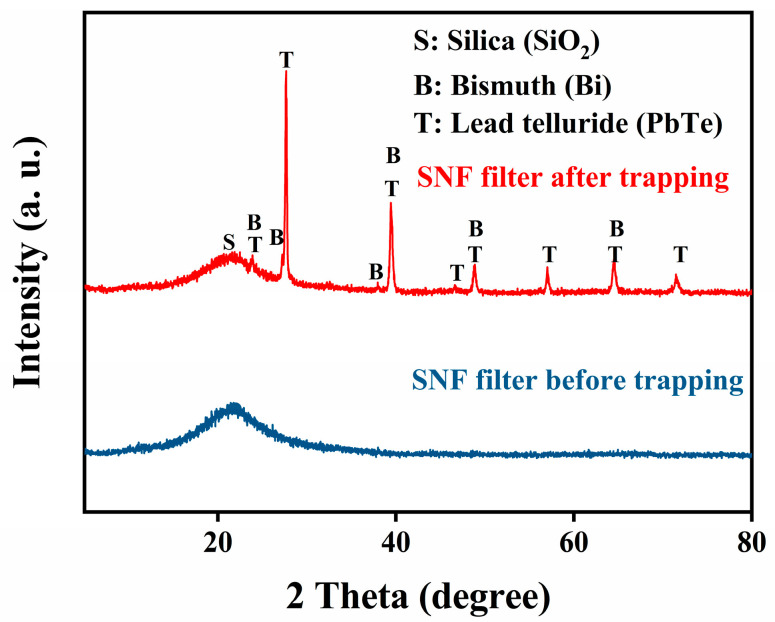
X-ray diffraction (XRD) patterns of the unused SiO_2_ nanofibers and the LBE-Te@SiO_2_ nanofibers.

The corresponding phase structures of the unused SiO_2_ nanofibers and the LBE-Te@SiO_2_ nanofibers were analyzed by XRD, as shown in Figure 2. The SiO_2_ nanofibers showed pure amorphous features with only a small envelope of diffraction peaks around 21°. In contrast, the XRD pattern of the obtained LBT-Te@SiO_2_ specimen revealed the characteristic reflections of an intermetallic PbTe phase around 23.8°, 27.6°, 39.4°, 46.6°, 48.8°, 57.0°, 64.4°, and 71.5°. The XRD patterns also showed the existence of a pure metallic Bi phase with diffraction angles of 27.2° and 37.9° and overlapping peaks with the PbTe phase at 23.8°, 39.4°, 48.8°, and 64.4°. No elemental lead or Te was detected from the XRD analysis. It is therefore strongly suggested that the Te that vaporized from the LBE was mainly in the form of PbTe, which to some extent confirmed that PbPo would be the main component in the cover gas in a real operating lead-based reactor [10,37,41,42].

**Figure 3:**
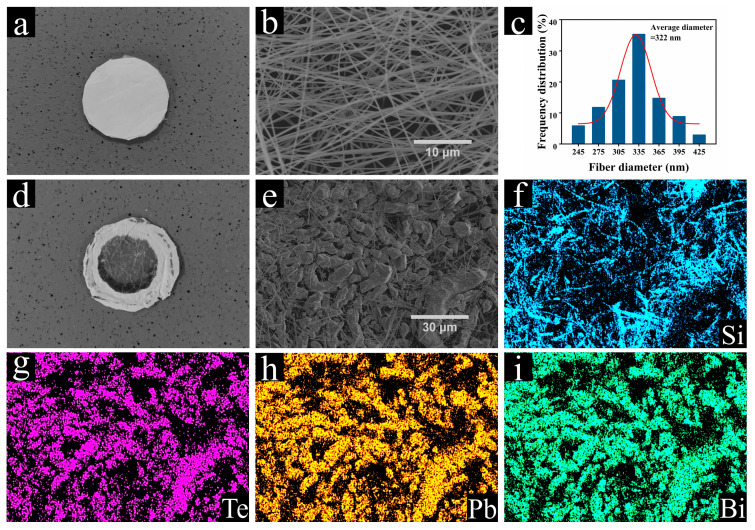
Photographs and scanning electron microscopy (SEM) images of SiO_2_ nanofiber membrane (**a**,**b**) before and (**d**,**e**) after the Te capture experiment at the filtration temperature of 400 °C, respectively. (**c**) Fiber diameter distribution of the SiO_2_ nanofibers and energy-dispersive X-ray spectroscopy (EDS) elemental mappings of (**f**) Si, (**g**) Te, (**h**) Pb, and (**i**) Bi are also presented.

By comparing the photographs of the SiO_2_ nanofiber membrane samples before and after the Te trapping experiment shown in Figure 3a,d, respectively, the successful capture of either Te, Bi, or Pb was realized. A closer SEM inspection of the samples before and after the experiment (Figure 3b,e, respectively) further proved this conclusion. As shown in Figure 3b, the unused SiO_2_ nanofiber membrane possessed well-distributed nanowires with uniform diameters. The nanofiber diameter was controlled to within 500 nm, and the average value was around 322 nm (Figure 3c). However, the sample morphology shown in Figure 3e reveals that the fiber membrane was significantly covered by a large quantity of larger particles, with sizes ranging from 0.5 to 50 µm. The elemental EDS mapping showed the uniformly distributed Te, Pb, Bi, and Si on the sample surface.

### 3.2. Factors Influencing Removal of PbBiTe by SiO_2_ Filter

#### 3.2.1. Filter Temperature

**Figure 4:**
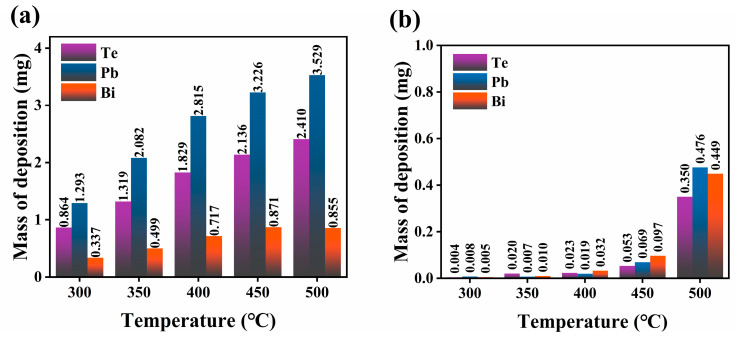
Obtained deposition masses of Te, Pb, and Bi from filters SNF1–SNF5 on (**a**) SiO_2_ nanofiber membrane and (**b**) stainless-steel mesh at different temperatures.

**Figure 5:**
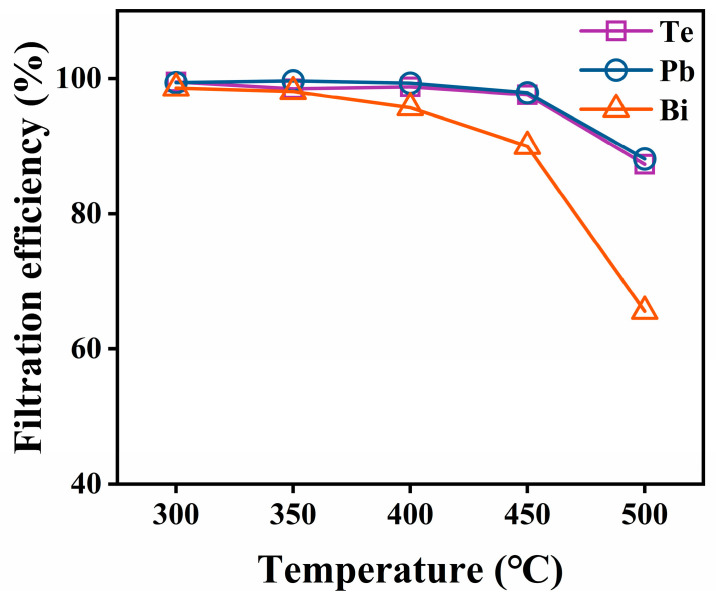
Filtration efficiencies of the SNF for Te, Pb, and Bi at different filtration temperatures.

ICP was used to measure the concentrations of volatile elements deposited on the filter after the capture experiment and to further calculate the deposition mass of each element on the filters. Figure 4 shows the determined masses of Te, Pb, and Bi deposited on the SiO_2_ nanofiber membrane and the stainless-steel mesh at different temperatures. As shown in Figure 4a, the deposited masses of all the elements on the SiO_2_ nanofiber membrane tended to decrease as the filtration temperature decreased. More vapor deposited on the tube surface before the vapor arrived at the filter when the filtration temperature was decreased. In addition, Figure 4b shows the captured masses of Te, Pb, and Bi on the stainless-steel mesh as a function of the filtration temperature. The results showed that the amounts of elements permeating through the SiO_2_ nanofiber membrane increased with the filtration temperature. In particular, when the filtration temperature changed from 450 °C to 500 °C, the penetration largely increased. Figure 5 shows the respective filtration efficiency of the SNF at different filtration temperatures. When the temperature was lower than 400 °C, the filtration efficiencies for Te, Pb, and Bi could be maintained at 98%, 99%, and 95%, respectively. However, when the temperature was higher than 400 °C, the filtration efficiency for each element began to gradually decrease. The filtration efficiencies for Te, Pb, and Bi dropped down to 85%, 86%, and 47%, respectively, when the temperature reached 500 °C. Among all the elements, the filtration efficiency of the SiO_2_ nanofiber membrane for Bi dropped most significantly. In summary, the removal of Te in the cover gas phase that evaporated from the liquid LBE alloy was affected by the trapping temperature.

The reasons for the decrease in the captured mass of the element of interest but the increase in the filtration efficiency of the SiO_2_ nanofiber membrane at a decreased filtration temperature can be explained as follows. At a lower filtration temperature, a larger temperature gradient was created between the filter and the vaporization source, which resulted in a large quantity of vapor deposition on the tube wall on the way from the source to the filter, and thus, a lower concentration in the gas phase. It is therefore evident that the captured masses of the elements were lower within a constant capturing timeframe. However, due to the identical and limited capturing capacities of the nanofiber membranes with the same sizes and porosities, a larger fraction of the element vapor could deposit on the membrane, and therefore, a higher filtration efficiency was achieved at a lower filtration temperature. Furthermore, a lower trapping temperature meant that the vapor could nucleate and grow on the nanofiber surface, even with a lower degree of subcooling, which also increased the nucleation rate and accelerated the deposition of vapor on the membrane surface. According to relevant studies, the vapor pressure of PbTe is between those of Pb and Bi, indicating that the volatility of PbTe is higher than that of pure Pb and lower than that of Bi [43,44,45,46]. Meanwhile, the melting point of Bi is much lower than that of PbTe; thus, a higher degree of subcooling can be achieved for PbTe. Consequently, capturing Bi is relatively more difficult than capturing PbTe, which results in a lower filtration efficiency for Bi.

#### 3.2.2. Loading Level of SiO_2_ Filter

**Figure 6:**
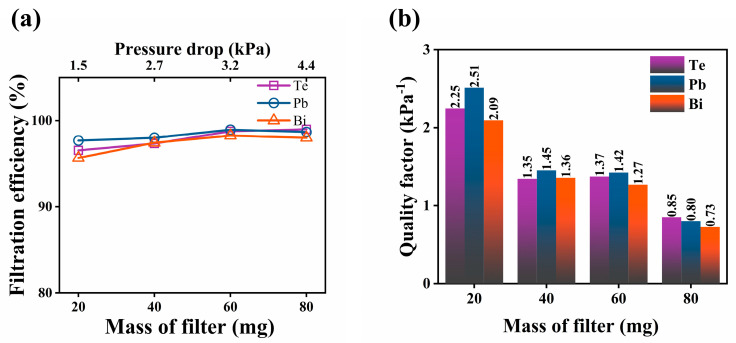
Comparisons of (**a**) filtration efficiency and (**b**) quality factor of the SiO_2_ filtration membranes with different SiO_2_ nanofiber loading levels for removing Te, Pb, and Bi at a filtration temperature of 400 °C.

Figure 6a shows the filtration efficiencies of the SNFs with different loading masses on the membrane for the removal of Te, Pb, and Bi at a filtration temperature of 400 °C. The corresponding samples used for this test were labeled as SNF-6, SNF-7, SNF-8, and SNF-9 with 20, 40, 60, and 80 mg loadings of SiO_2_ nanofibers, respectively. More generally, an area-specific loading density, defined as the loading mass divided by the cross-sectional area of the filter, can also be adopted as a characteristic structural parameter for the filter design. In this study, the corresponding area-specific loading densities of the filters SNF-6–SNF-9 were calculated to be around 0.76, 1.51, 2.27, and 3.03 mg/cm^2^, respectively.

The results indicate that the filtration efficiency gradually increased as the loading level or the area-specific loading density of the SiO_2_ nanofibers increased. However, higher filtration efficiencies of 96%, 97%, and 95% could still be achieved for Te, Pb, and Bi, respectively, even with the lowest filter loading (20 mg) in the current experiment. In general, the increase in the loading level of the SiO_2_ nanofibers would not change the pore size and distribution on the membrane surface. Both the porosity and tortuosity of the obtained membranes remained almost the same for all the SiO_2_ nanofiber membranes in the current experiment. However, a higher loading mass of the SiO_2_ nanofibers in a membrane would inevitably increase the thickness of the membrane so that the penetration pathway of the gas phase through the membrane would increase. This provided a greater chance for the membrane to capture the elements of interest in the current study.

However, this does not mean that a continuously increased loading level of the SiO_2_ nanofibers would always be a better choice. One must consider the pressure drop for the operation. An increase in the membrane thickness would cause a significantly increased flow resistance. More importantly, the experiment revealed that the majority of the vapor deposition occurred at the front surface (feed side) because of the increased surface area due to the accumulated deposits, which decreased the available pore size on the surface for vapor penetration, as evidenced in Figure 3e. Figure 6a shows that the increase in filtration efficiency became insignificant when the loading level of the SiO_2_ nanofibers exceeded 60 mg. The calculated quality factor (QF) for the SiO_2_ nanofiber membranes is shown in Figure 6b. The quality factor of the membrane decreased with the increase in the area-specific loading density of the SiO_2_ nanofibers. Significant changes occurred between the SNF-6 and SNF-7 filters and between the SNF-8 and SNF-9 filters. The filtration efficiencies did not increase significantly from SNF-6 to SNF-9, and thus, the apparently decreased QF indicated a significant increase in the pressure drop when the SiO_2_ loading changed from SNF-6 to SNF-7 and from SNF-8 to SNF-9. In particular, the pressure drop increased by 68.8% when the filter loading level changed from SNF-8 to SNF-9. Meanwhile, the QF value did not vary significantly for SNF-7 and SNF-8, with different SiO_2_ nanofiber loadings. In summary, an area-specific loading density between 1.51 and 2.27 mg/cm^2^ may be the optimal SiO_2_ loading level for such nanofiber membranes to achieve the best overall filtration performance to remove Te, Pb, and Bi vapors in the lead-based reactor.

#### 3.2.3. Flow Rate of Carrier Gas

**Figure 7:**
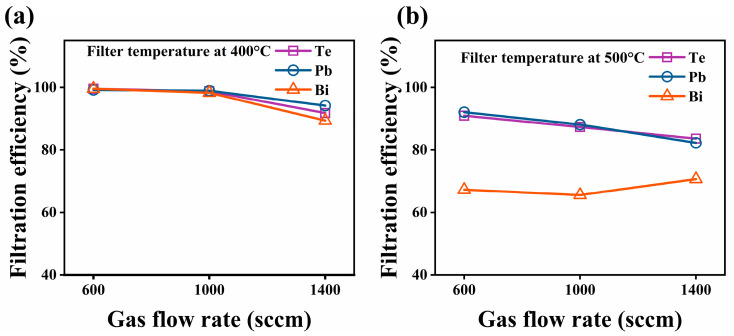
Filtration efficiencies of the SNF for Te, Pb, and Bi at different gas flow rates at filtration temperatures of (**a**) 400 °C and (**b**) 500 °C.

To study the filtration efficiency under different gas flow conditions, flow rates of 600, 1000, and 1400 sccm were selected for testing. The filtration efficiency results of the SNF at different gas flow rates and filtration temperatures of 400 °C and 500 °C are shown in Figure 7. The results revealed that the increase in the gas flow rate generally reduced the efficiency of the filter to remove aerosols in the gas phase. The temperature effect of the reduced filtration efficiency at 500 °C compared to that at 400 °C under the same gas flow rate was consistent with the findings in Figure 5. All these results suggest that a high gas flow rate and filtration temperature are not recommended for the removal of gaseous components and aerosols in the gas phase by the SiO_2_ fiber filter. In particular, increasing the gas flow rate would shorten the contact time of the vapor or clusters with the fiber surface and ultimately affect the adhesion and aggregation on the surface, thereby lowering the filtration efficiency of the SiO_2_ nanofiber membranes for Te, Pb, and Bi in the gas phase.

#### 3.2.4. Cycle-Use Performance of SiO_2_ Nanofiber Filter

**Figure 8:**
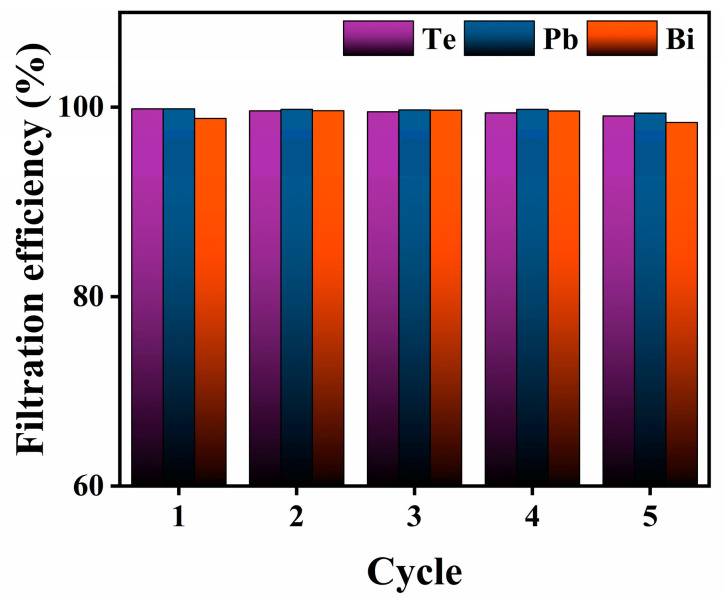
Filtration efficiency in five cycles.

The cycle-use experiments were performed at a filtration temperature of 400 °C and heated for 5 h. The elements of Te, Pb, and Bi on the SiO_2_ nanofiber membrane were desorbed by nitric acid solution (7 mol/L). Then, the filter membrane was freeze-dried to obtain a regenerative filter for repeatable experiments. After repeated filtration for five runs, the filtration efficiency of SNF did not decrease significantly (Figure 8). The results showed that the filtration performance of the SiO_2_ filter had excellent cycle-use performance, which ensures the long-term stability of purification system at high temperatures.

### 3.3. DFT Calculation

**Figure 9:**
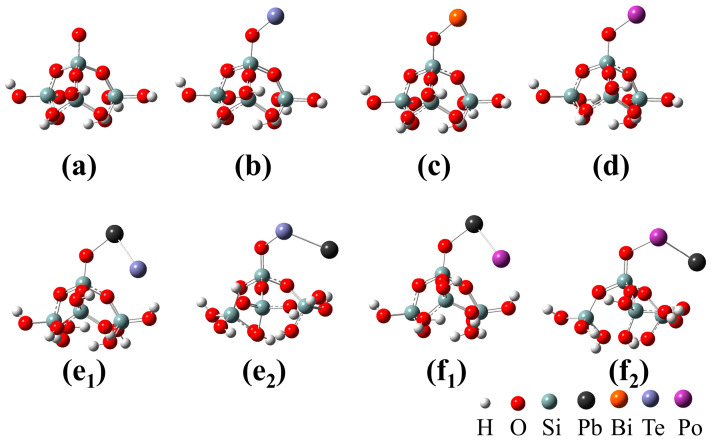
Adsorption configurations of monoatomic molecules (Po, Te, and Bi) and diatomic molecules (PbTe and PbPo) on the ≡Si-O adsorption site used for density functional theory (DFT) calculations: (**a**) SiO_2_, (**b**) Te/SiO_2_, (**c**) Bi/SiO_2_, (**d**) Po/SiO_2_, (**e_1_**,**e_2_**) PbTe/SiO_2_, and (**f_1_**,**f_2_**) PbPo/SiO_2_.

The adhesion behaviors of the volatile components PbTe and Bi from the LBE-Te sample with amorphous SiO_2_ were considered. For comparison, the adhesion properties of Te and Po-containing molecules (Po, PbPo)) were also evaluated. Figure 9 shows the adhesion configuration of monoatomic molecules (Po, Te, Bi) and diatomic molecules (PbTe, PbPo) at the defect sites on the amorphous SiO_2_ surface. Table 2 lists the calculated adhesion energies of the different configurations on the amorphous SiO_2_ surface defect sites. The results showed that the adhesion energies of all the configurations were negative, indicating that the molecules could be spontaneously adsorbed on the SiO_2_ surface. The adhesion energies for monoatomic molecules (Te, Bi, and Po) on the SiO_2_ surface were between −3.72 and −4.34 eV, while the adhesion energies for diatomic molecules (PbTe and PbPo) on the SiO_2_ surface ranged from −2.0 to −2.96 eV. The adhesion of monoatomic molecules was significantly stronger than that of the diatomic molecules on the SiO_2_ surface. However, Te molecules that had strong interactions with SiO_2_ were not found in the current Te capture experiment, indicating that the volatile component of Te in the LBE was mainly PoTe. This suggested that the most likely component to contain Po in the cover gas in a real reactor will be PbPo. The calculated adhesion energy results based on different adsorption configurations for diatomic components revealed that configurations e_1_ and f_1_, with the Pb atom in the PbTe or PbPo molecules binding to the SiO_2_ defect sites, had stronger adhesion effects than configurations e_2_ and f_2_, respectively. This indicated that lead dominated the interactions between PoPb and the filter materials. Based on the experimental and theoretical results, PbTe and Bi were removed from the gas by strong adhesion to the surfaces of the SiO_2_ nanofibers. Nonetheless, the filtration efficiency of the SNF for PbTe was significantly better than that for Bi at filtration temperatures above 350 °C, which was inconsistent with the calculation result that the adhesion energy of Bi was greater than that of PbTe. This was because the melting point of Bi was much lower than the filtration temperature used in this study (300–500 °C), which increased the difficulty for capturing Bi.

## 4. Conclusions

In this work, electrostatic spun flexible SiO_2_ nanofiber membrane filters exhibited outstanding high temperature and corrosion resistance. This paper investigates the filtration performance of SiO_2_ nanofiber membrane filters under different conditions of filtration temperature, loading level of the SiO_2_ filter, and flow rate of carrier gas. In general, increasing the filtration temperature and the flow rate of carrier gas has a negative effect on the filtration efficiency of SNF. For example, the filtration temperature and the flow rate of carrier gas in this experiment are not suitable to exceed 400 °C and 1000 sccm. Similarly, the load level of SiO_2_ filters should not be too excessive to avoid large pressure drops in the filtration system. Due to its good mechanical properties and acid resistance, the flexible SiO_2_ nanofiber membrane could maintain its filtration performance after multiple regenerations. The strong adhesion of amorphous SiO_2_ to the volatile components in LBE-Te was demonstrated by DFT calculations. Based on the filtration efficiency of SiO_2_ nanofiber membranes for Te aerosols up to 99%, SiO_2_ nanofibers are expected to be used as a filtration material to remove Po from lead alloy-cooled reactors.

## Figures and Tables

**Table 1:** Experimental conditions for different aerosol capture experiments.

Experiences	Test No.	Mass of Nanofibers (mg)	Trapping Temperature (°C)	Flow Rate (mL/min)
Set I	SNF-1–SNF-5	60	300–500	1000
Set II	SNF-6–SNF-9	20–80	400	1000
Set III	SNF-10–SNF-12	60	400	600–1400
Set IV	SNF-13–SNF-15	60	500	600–1400
Set V	SNF-16	60	400	1000

**Table 2:** Calculated adhesion energies of monoatomic molecules and diatomic molecules on the ≡Si-O· adsorption sites.

Diatomic Molecules	Configuration	*E*_ads_ (eV)	Monatomic Molecules	Configuration	*E*_ads_ (eV)
Te	b	−4.34	PbTe	e1	−2.83
Bi	c	−3.72		e2	−2.00
Po	d	−4.15	PbPo	f1	−2.96
				f2	−2.06

## Data Availability

Not applicable.
